# Proximity to death and health care expenditure increase revisited: A 15-year panel analysis of elderly persons

**DOI:** 10.1186/s13561-019-0224-z

**Published:** 2019-03-11

**Authors:** Viktor von Wyl

**Affiliations:** Epidemiology, Biostatistics & Prevention Institute, University of Zurich, Hirschengraben, 84, CH-8001 Zurich, Switzerland

**Keywords:** Health care expenditures, Ageing, Proximity-to-death, Morbidity, Switzerland, H51, J11, I10

## Abstract

**Background:**

Health care expenditures (HCE) are known to steepen with increasing age, but the contributions of biological age, morbidity, or proximity to death as cost drivers are debated. Age-associated HCE growth can be studied across two dimensions: within fixed groups of persons with the same birth year followed over time (birth cohort), or the same age classes (e.g. 66 to 70 year olds) at different time points (age-class analysis). Using health insurance claims data including morbidity and mortality information, HCE growth was analyzed in Swiss mandatory health insurance for the years 1996 to 2011 and compared across the two age dimensions.

**Results:**

Deflated HCE were analyzed for 104,000 persons from three birth cohorts (1921-25, 1926-30, 1931-35). Two-part regression models were adjusted for proximity-to-death (death within same or next calendar year) and morbidity indicators (hospitalization, high drug expenditures, and pharmaceutical cost groups from 2006 onwards).

When analyzing HCE growth within birth cohorts, controlling for survival and morbidity status decreased age-associated HCE estimates by 31% to 51% compared to crude age averages. The total HCE volume share of decedents rose from 19% to 31% in the 1931-35 birth cohort and from 28% to 51% for the 1921-25 birth cohort.

The analysis of same age classes (e.g. 71-75 year olds) over different years revealed no HCE growth (steepening) in excess of deflation for groups aged 75 years or less, and only moderate HCE growth for those ≥76 years. For the 76+ age classes, the population fraction of decedents decreased by -3% (age 76-80) and -15% (age 81-85) over time, whilst the total HCE volume share of decedent-associated HCE increased by +16% and +9%, with an HCE growth of +3.2% and +2.5% per year.

**Conclusions:**

HCE growth was dominated by end-of-life HCE, but residual age-associated HCE growth remained pertinent, the extent of which however depended on morbidity indicator definitions. A better understanding of shifts in chronic disease prevalence with rising age, as well as associated HCE and survival impacts of treatment will be key for further refining future HCE projections.

**Electronic supplementary material:**

The online version of this article (10.1186/s13561-019-0224-z) contains supplementary material, which is available to authorized users.

## Introduction

The question whether age is a true driver of health care expenditure (HCE) growth has substantial societal relevance because it is feared that demographic aging, defined as an increasing share of elderly persons and a rising average age, may bring many social health insurance systems to their limits. Yet, the relationship between aging and HCE growth is not entirely understood. For example, the “red herring theory” postulates that the association between rising age and HCE growth is actually driven by proximity-to-death. [1] If true, this would imply that additional life years gained would be spent in relatively good health and therefore not automatically trigger an accelerated overall HCE growth. [2] By contrast, if medical treatments among elderly were to prolong lifespan but also lead to a lifelong dependency on follow-up care, then population aging may indeed contribute to substantial further HCE growth. Given this range of possible scenarios, a better understanding of the relationship between aging and HCE growth is key in order to tackle future challenges of demographic aging in health care systems.

Using longitudinal, individual-level Swiss health insurance claims data from more than 104,000 elderly (>60 years), the present study aimed to dissect the impact of proximity-to-death on general HCE growth over a time period of 15 years The availability of this complete panel data set (including morality data) allowed analyses of the data in two age/time dimensions. First, analyses within birth cohorts allowed deeper insights into drivers for age-associated HCE growth, whereas HCE comparisons across specific age classes in different calendar years (e.g. HCE of 61-65 year olds in 1997 and 2001) helped to acquire a better understanding of broader patterns of health status and care utilization over time. Thereby, the findings are contributing to ongoing scientific debates regarding identification of HCE drivers in aging societies (analysis 1), whilst the second analysis addressed the as-of-yet unsolved question whether HCE growth is truly more pronounced in older age classes as postulated by some studies. [3, 4]

This manuscript is structured as follows: The next section discusses the database and the analytic strategy. The results section starts with a description of the study sample (with emphasis on HCE and mortality rates) and proceeds with a presentation of regression analyses results and an analysis of HCE growth rates for different sub-populations defined by morbidity status and proximity-to-death. The paper closes with a discussion section and remarks on study strengths and caveats for interpretation.

## Methods

### Literature Search

Overall, the notion of rising HCE with increasing age is uncontested [5, 6]. Nevertheless, opinions diverge on the main causes of age-associated HCE growth. Possible drivers reported in the literature are, for example, shifts in morbidity-related factors, technological innovations, and proximity-to-death. This latter aspect has gained prominence as the “red herring hypothesis”, put forth by Zweifel and colleagues. [1, 7] Based on their empirical analysis of HCE data from survivors and decedents, the authors observed that the relevance of age parameters diminished once variables for time-to-death were included in the models. This finding led to their claim that aging is in fact a distraction from the true causes of age-related HCE increases, with the real reason being the increasing proximity-to-death. Their work has sparked an ongoing debate on, and long line of studies investigating, drivers of age-related HCE growth.

The existing literature can broadly be grouped into micro-econometric analyses of HCE data (e.g. from health insurers), which were testing the statistical significance of time-to-death and aging parameters in regression models (mostly two-part regressions) for various HCE components (e.g. long-term care or prescription drug use) and by employing differing morbidity adjustments. [7–10] A second strand of research approached the question from a macro perspective via a combination of micro-data analyses and population-level simulations of HCE growth [11–16]. The majority of both study types seem to acknowledge a major role of proximity-to-death as HCE driver, but some diverge in their assessment of whether there is room for aging as an additional, relevant factor. [14] More recently, the scientific debate has shifted towards clarifying the role of morbidity viz. proximity-to-death. Indeed, some authors maintain that proximity-to-death is in fact a proxy for morbidity status. [10, 17]

The topics of demographic aging and rising HCE are also pertinent for Switzerland. Currently, elderly persons aged 65 and older account for 43% of all HCE accrued in mandatory health insurance, and projections foresee a rise in the percentage of elderly from 18% in 2015 to 26% by the year 2045 [18], with significant implications for HCE growth [15]. Several Swiss studies have already looked into the relationship between HCE increases and rising age. For example, by analyzing insurance claims data, Felder and colleagues have repeatedly found that proximity-to-death is a much stronger determinant for cost increases in health care than age [1, 7, 8]. Along the same lines, Steinmann and colleagues confirmed a dominant role of proximity-to-death but further also emphasized the need for distinction between mortality- and morbidity-related costs in the context of HCE growth predictions [19]. This view is challenged by Colombier [14] who performed cost projections informed by long-term macro-data analysis. These analyses maintained strong age-dependencies and hence predicted substantial cost growth in the health care sector as a result of population aging in Switzerland [14]. Along the same lines, as a result of demographic aging and higher HCE expenditure growth in older age groups, von Wyl and Beck predicted a substantial intergenerational shift in the burden of HCE from older to younger age groups, which is mediated through the redistributive effects of risk adjustment in mandatory health insurance [20].

The present analysis extends these findings by further investigating the role of morbidity and proximity-to-death as drivers for the previously observed higher HCE growth in older age groups over a 15 year period. Moreover, an additional novelty of this manuscript is the two-dimensional growth analysis within birth cohorts and across age classes at different time points, which allows a dissection of cohort- and calendar-year specific contributions to cost growth.

### Study objective

**Fig. 1:**
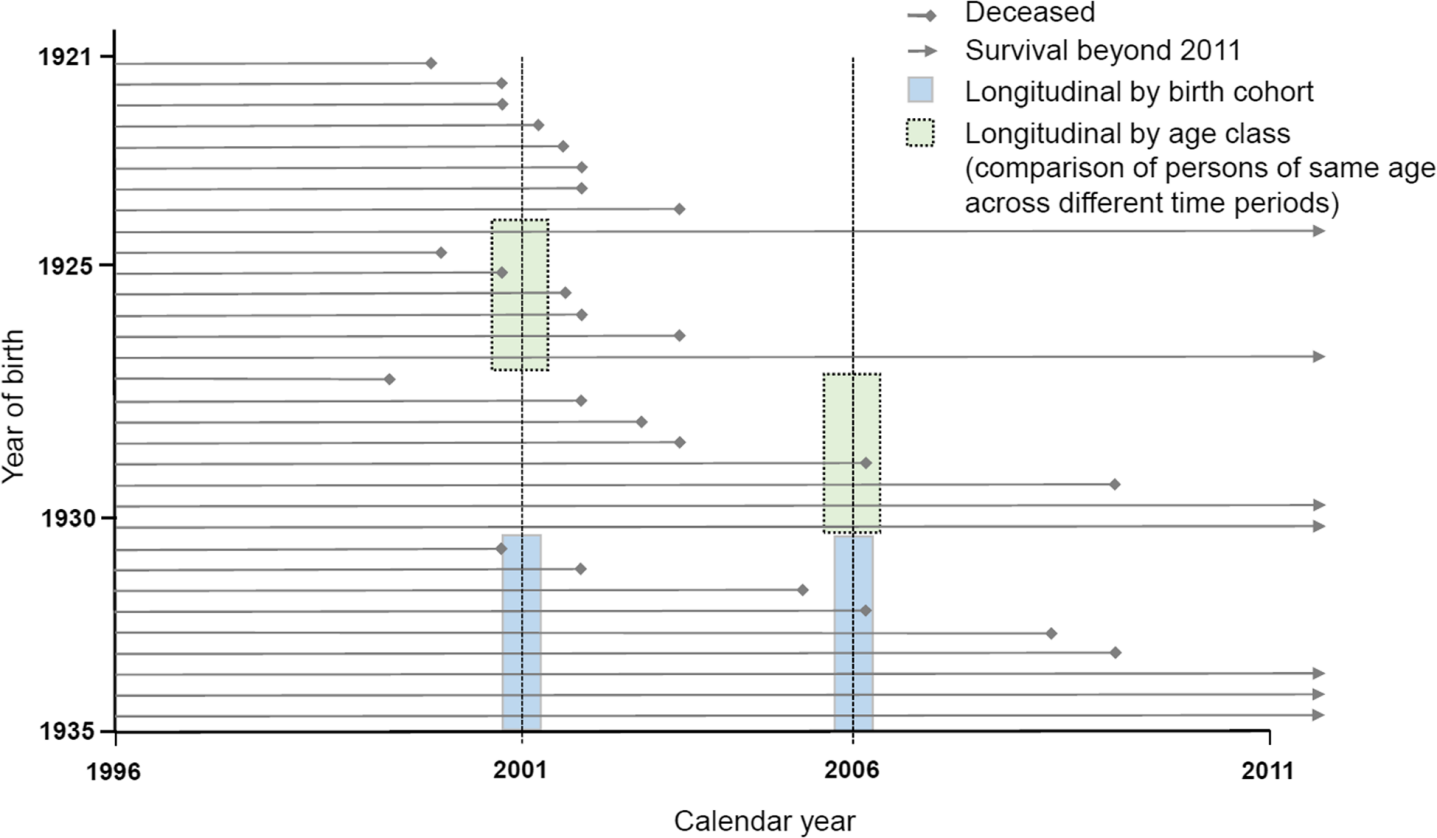
Schematic depiction of study design

The main objective of this study was to assess the impact of proximity-to-death and morbidity (as defined by the three proxy measures introduced below) on HCE over time. The analysis comprised of three steps. The birth cohort analysis aimed at revealing age-associated HCE growth patterns over a duration of 15 years (Fig. 1, blue boxes). By contrast, the age class analysis focused on the evolution of HCE within specific age classes over time (e.g. the groups of 71-75 year olds insured in the years 1996 to 2006; Fig. 1, green boxes). Results from this analysis were indicative of more general patterns of health care consumption, and possibly also of age-specific health status changes over time. Finally, the decomposition step employed results from the “birth cohort” and the “age class” analyses in order to assess the importance of different drivers of HCE growth, that is, of proximity-to-death, morbidity indicators, as well as unspecified, residual age-associated factors at the population level.

### Data Sources

This study used individual-level HCE data from the Swiss social health insurance, which is mandatory for all persons living in Switzerland and offers comprehensive coverage of all essential medical in- and outpatient treatments. Anonymized data was provided by CSS Insurance, the largest, nationwide operating Swiss health insurer, which has a market share of approximately 16%. The data were reimbursement claims covering a 15-year period (from 1996 to 2011). Because costs for inpatient hospital stays are shared evenly between cantons and insurers, they were doubled for the purpose of this analysis. The database contained annualized HCE per insured, as well as the coverage duration for a given year (ranging from 1 to 12 months). To account for partial coverage years (e.g. due to death), yearly HCE were rescaled to monthly HCE by dividing total annual HCE by coverage months.

All HCE were deflated on the basis of overall cost growth in Swiss mandatory health insurance. The deflation factors were derived from data on Swiss risk adjustment and included annual HCE growth rates for all adult insured between 18 to 60 years of age (on average 3.3% per annum).

The population included in this study consisted of persons who were born between 1921 and 1935. For the results presentation, these individuals were grouped into three birth cohorts of persons born between 1921-1925, 1926-1930, and 1931-1935 (i.e. persons aged 71 to 75, 66 to 70, and 61 to 65 at baseline in 1996, respectively), whose survival status and HCE were studied over the 15-year observation period. The study population was restricted to persons with at least 12 months of total follow-up and insurance coverage until death or the full observation period.

### Variables

**Fig. 2:**
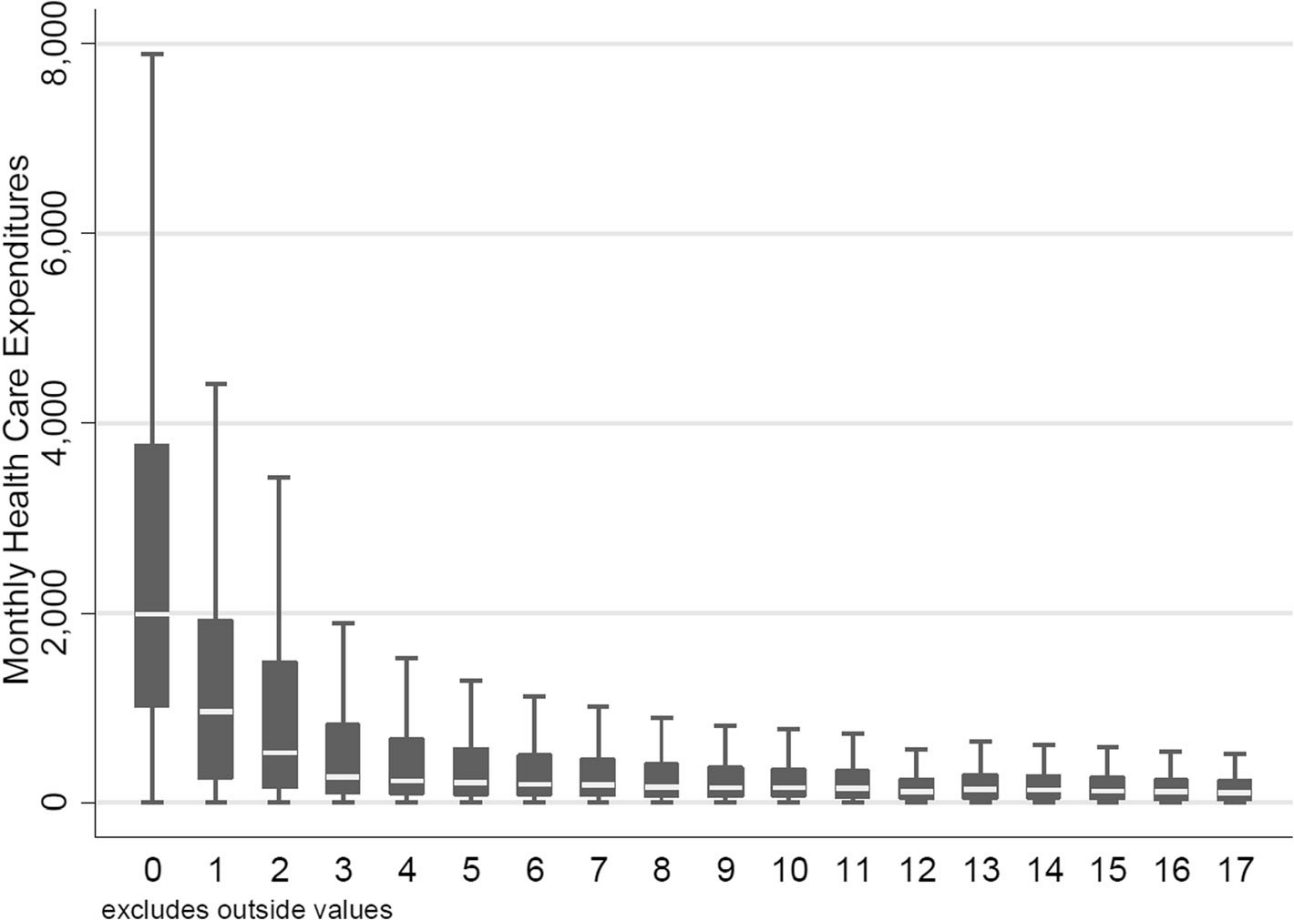
Crude monthly HCE, by survivor status. The x-axis represents years to death, whereby 0 stands for death in same calendar year, 1 equals death in following year, etc

Swiss insurers collect demographic information (age, gender), as well as the year of death (although not the cause). Mortality information was available up until the year 2013. Proximity-to-death was defined as years until the event, with 0 indicating death in the same calendar year, 1 in the following calendar year, etc. For the analysis, proximity-to-death greater than 2 years were censored (i.e. set to 2 years) because HCE dropped markedly beyond 2 years prior to death and remained at approximately similar levels (Fig. 2). Larger censoring cut-offs (3 years and more) were explored in sensitivity analyses but did not materially alter results (not shown).

In Switzerland, outpatient diagnostic information are not routinely available to health  insurers (and inpatient diagnoses only since 2012). However, the data contained information on hospitalizations, as well as nursing home stays and amounts of outpatient drug expenditures, which allowed emulation of some of the morbidity indicators used by Swiss risk adjustment over the full observation period (see below, [21, 22]). In particular, these morbidity indicators record whether a person had nursing home stays or inpatient hospitalization in the prior calendar years, as well as high medication costs above a pre-defined threshold in the previous calendar years. These indicators were developed, validated, and are implemented in the Swiss risk adjustment scheme and strike a compromise between ease of estimation and predictive power. For the purpose of this study, high medication costs were defined as outpatient prescription drug costs above CHF 2750 at baseline. For subsequent analysis years, this threshold was adjusted by yearly HCE cost growth (amounting to approx. CHF 5000 in 2015, which corresponds to the threshold used in Swiss risk adjustment). The only diagnostic information were pharmaceutical costs groups (PCG), which were available in this dataset from 2006 onwards. These PCG are derived routinely from outpatient drug prescriptions for 21 chronic illnesses that require continuous treatment with disease-specific medications (such as those for HIV, Diabetes, or depression). [21, 23]

### Analysis Strategy

Multivariable regression analyses of increasing complexity were employed to factor out the effects of imminent death (i.e. within the same or next calendar year) and morbidity (as indicated by hospitalizations, nursing home stays, or high outpatient drug expenditures in the prior calendar year).

For the regression analyses, average monthly health care expenditures in Swiss Francs (1 CHF equaled 0.86 Euro or 1 US$ in July 2018) were extracted for the years 1997 through 2011 and analyzed by means of two-part models (using robust standard errors). The two-part models consisted of a logit-model part for the probability of having >CHF 0 health care expenditures in a given period. The second part consisted of a generalized linear model (glm) for the health care expenditure amount of persons with positive HCE. Based upon exploratory analyses and comparisons of goodness-of-fit criteria (Aikaike’s Information Criterion, AIC), the generalized gamma method was chosen for all two-part regression implementations in this study (not shown).

The two-part regression framework used in this study can be described by the following two equations for the basic model

$$ \Pr \left( HCE>0\right)= sex+ baseline\  age+ baseline\ {age}^2+ calendar\ year\ dummies+\left( baseline\  age\ast calendar\ year\ dummies\right)+\left( baseline\  age\ast sex\right) $$

(which is the logit part) and

$$ \Pr \left(\overline{HCE}\right| HCE>0\Big)= sex+ baseline\  age+ baseline\ {age}^2+ calendar\ year\ dummies+\left( baseline\  age\ast calendar\ year\ dummies\right)+\left( baseline\  age\ast sex\right), $$

representing a generalized linear model (glm) with log link and gamma distribution.

The following variables and attributes were analyzed: sex (male/female), age at baseline as a continuous variable, and calendar years as dummy variables. The interaction terms were chosen a priori based on existing literature (e.g. [10, 17, 24]).

Model specifications of the logit and glm parts for the mortality- (i.e. proximity-to-death) and morbidity-adjusted model were as follows:

$$ \Pr \left( HCE>0\right)\left(\overline{HCE}\right| HCE>0\Big)= sex+ baseline\  age+ baseline\ {age}^2+ calendar\ year\ dummies+ hospitalization+ nursing\ home\ stay+ high\ drug\ expenditures+ time\ to\ death $$

as well as two-way interactions terms baseline age * sex, baseline age * calendar years, baseline age * hospitalization in prior year, time to death * hospitalization in prior year, baseline age * time to death, and calendar years * time to death. Again, interaction terms were chosen a priori based on existing literature and subject knowledge about cost trajectory shapes of end-of-life HCE in Switzerland. [6]

A third regression model considered only mortality adjustments but no morbidity-related variables and interaction terms. Moreover, in a sensitivity analysis the full mortality- and morbidity-adjusted model was complemented with PCGs, which were available from 2006 onwards.

The two-part estimation was performed on logarithms of HCE, and regression model estimates were then back-transformed into Swiss Francs according to the following equation (for individual *i*):

$$ \left({\overline{HCE}}_i\right|\ {x}_i\left)=\Pr \left({HCE}_i>0\right|\ {x}_i\right)\times \left({\overline{HCE}}_i\right|{HCE}_i>0,{x}_i\Big) $$

Confidence Intervals for predictions on the original HCE scale were estimated via bootstrapping with 500 replications. For presentation, the model results were aggregated over three birth cohorts [1921-1925, 1926-1930, and 1931-1935] to allow for the longitudinal and cross-sectional comparisons outlined in Fig. 1. Along the same lines, calendar time was available in full for regression models but is – for ease of interpretation - illustrated only for four calendar years that are approximately 5 years apart (1997, 2001, 2006, 2011), in order to ensure a full shift of all age classes into the next higher category. The year 1997 was chosen as the baseline year instead of 1996 because some analyses use morbidity indicators that are based on 1-year lagged information, which were not available in this dataset before 1996.

In a final step, it was explored as to what extent imminent death may act as a driver of HCE growth across different age groups. The general approach was to group all insured into three hierarchical groups in a given reference year: those who died within two years (*s* ≤ 1), those with morbidity indications but survival beyond two years (*s* > 1, *m* = 1), and those without morbidity indicators and survival beyond two years (*s* > 1, *m* = 0). Next, each group’s share of the overall cost volume in a reference year was calculated according to the following equation, with *n* denoting number of individuals and $\overline{HCE}$ representing regression coefficients.

The total HCE volume *V* in a given year y for a given birth cohort b is defined as follows.

$$ {V}_{b,y}={n}_{b,y,\kern0.5em s\le 1,m\in \left\{0,1\right\}}\ast {\overline{HCE}}_{b,y,s\le 1,m\in \left\{0,1\right\}}+{n}_{b,y,s>1,m=1}\ast {\overline{HCE}}_{b,y,s>1,m=1}+{n}_{b,y,s>1,m=0}\ast {\overline{HCE}}_{b,y,s>1,m=0} $$

The contributions *F* of each group to cost volume increase is further calculated by

(for decedents)

$$ {F}_{b,y,s\le 1,m\in \left\{0,1\right\}}=\left[{n}_{b,y,s\le 1,m\in \left\{0,1\right\}}\ast {\overline{HCE}}_{b,y,s\le 1,m\in \left\{0,1\right\}}\right]/{V}_{b,y} $$

(for persons with morbidity indicators)

$$ {F}_{b,y,s>1,m=1}=\left[{n}_{b,y,s>1,m=1}\ast {\overline{HCE}}_{b,y,s>1,m=1}\right]/{V}_{b,y} $$

and (for persons without morbidity indicators)

$$ {F}_{b,y,s>1,m=0}=\left[{n}_{b,y,s>1,m=0}\ast {\overline{HCE}}_{b,y,s>1,m=0}\right]/{V}_{b,y} $$

All models and calculations were implemented using Stata 13 and the stpm package. [25]

## Results

### Descriptive analyses of the study sample and HCE

The full database contained information from approximately 125,000 individuals. However, the selection criterion led to the exclusion of 17% of individuals who either switched insurance carriers or did not have 12 months of follow-up time. Excluded persons were more likely males but did not differ significantly from included persons with respect to HCE distribution (median CHF 125 [interquartile range 32; 331] per month for excluded vs. CHF 123 [41; 295] for included persons, rank-sum p-value 0.195).

The final sample contained information of 104,215 Swiss insured who were at least 61 years old at baseline. As expected, women were in the majority (59%). Furthermore, the birth cohort distribution looked as follows. In 1997, 38% were between 61 to 65 years old (1931-1935 birth cohort), 33% were aged between 66 and 70 (1926-1930 birth cohort), and 30% were between 71 to 75 years old (1921-1925 birth cohort). Of those, 66,175 individuals remained in the analysis until 2011, and 40% of all individuals died during the observation period (not shown), with statistically significant differences across birth cohorts (Additional file 1: Figure S1) and/or morbidity status (not shown).

**Fig. 3:**
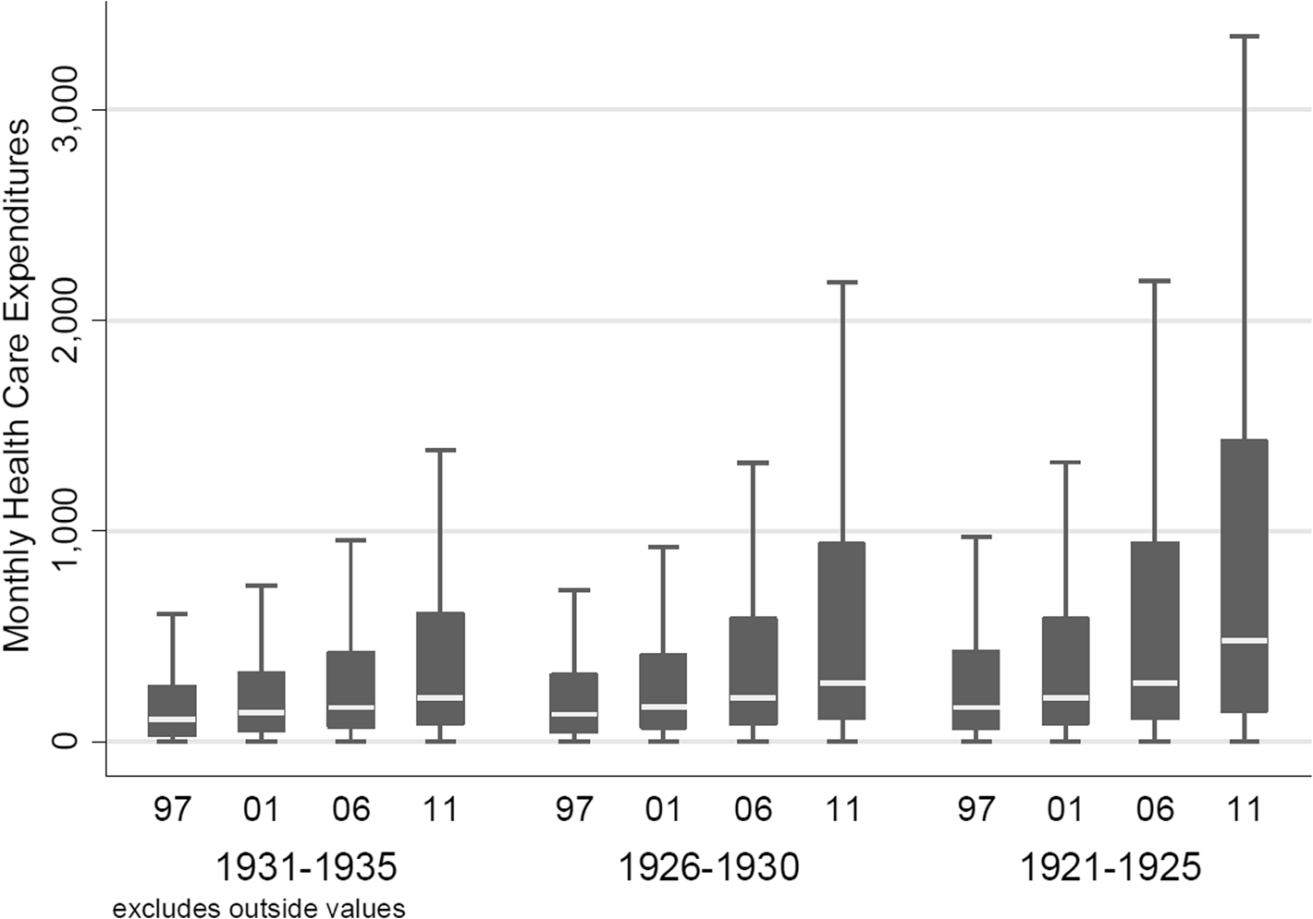
Crude monthly HCE plotted per calendar year (inner x-axis labels) and by birth cohort, that is, the same individuals followed over time (outer x-axis labels)

**Fig. 4:**
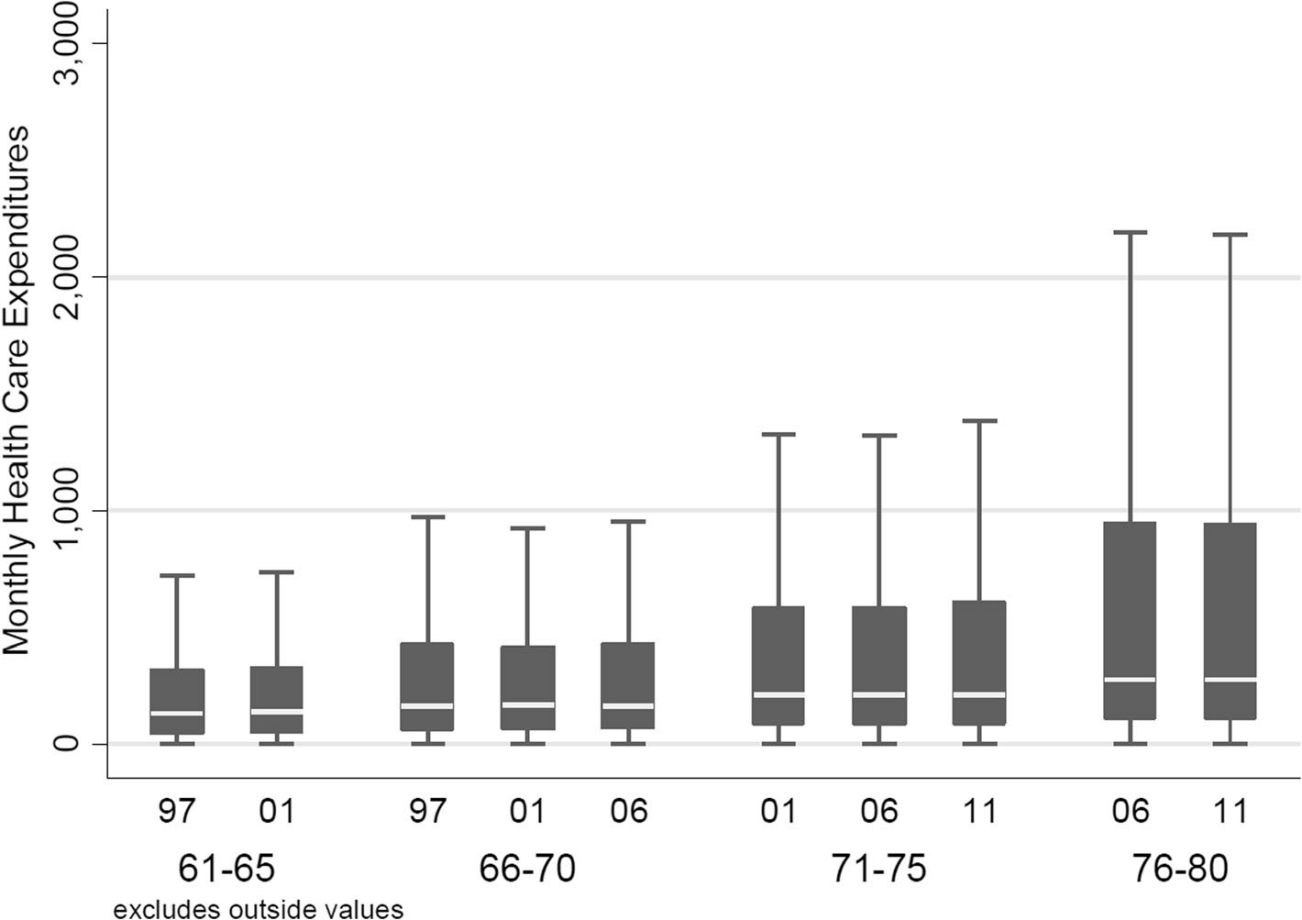
Crude monthly HCE per year (inner x-axis label) and by age class, that is, age classes held constant over different time periods (outer x-axis label). Note that each age class includes individuals from different birth cohorts

Table 1 illustrates averages of deflated monthly HCE, which increased markedly in the presence of morbidity indicators and with increasing proximity-to-death, as well as rising calendar year. The raw data displayed in Table 1 are complemented by box-and-whisker plots of monthly HCE by proximity-to-death (Fig. 2), over time by birth cohort (Figure 3), and by the same age classes over time (Fig. 4). Figures 2 and 3 confirm the expected relationships between rising HCE and proximity-to-death and increasing age, respectively. By contrast, Fig. 4 shows results for constant age classes over time and indicates almost no (deflation-indexed) HCE changes over calendar time. However, HCE increased across age classes as expected, with higher HCE as age increases.

**Table 1:** Descriptive analyses of HCE, stratified by remaining lifetime and analysis year (stratified by survivor status and presence of morbidity indicators as defined for the mortality and morbidity adjusted model)

Year	Subgroup	N	HCE mean (SD)	Females (%)	1931-1935	1926-1930	1921-1925
1997	All	104215	411 (1013)	61794 (59.3%)	38522 (37%)	34758 (33.4%)	30935 (29.7%)
1997	Survivor, no morbidity	82540	246 (571)	49973 (60.5%)	32008 (38.8%)	27617 (33.5%)	22915 (27.8%)
1997	Survivor, with morbidity	17472	747 (1196)	10056 (57.6%)	5583 (32%)	5766 (33%)	6123 (35%)
1997	Deceased	4203	2260 (2944)	1765 (42%)	931 (22.2%)	1375 (32.7%)	1897 (45.1%)
2001	All	95469	455 (962)	58022 (60.8%)	36554 (38.3%)	31914 (33.4%)	27001 (28.3%)
2001	Survivor, no morbidity	71247	265 (500)	44175 (62%)	29082 (40.8%)	23840 (33.5%)	18325 (25.7%)
2001	Survivor, with morbidity	19383	742 (1017)	11647 (60.1%)	6328 (32.6%)	6601 (34.1%)	6454 (33.3%)
2001	Deceased	4839	2104 (2619)	2200 (45.5%)	1144 (23.6%)	1473 (30.4%)	2222 (45.9%)
2006	All	82424	595 (1199)	51821 (62.9%)	33582 (40.7%)	27808 (33.7%)	21034 (25.5%)
2006	Survivor, no morbidity	57562	325 (623)	36794 (63.9%)	25484 (44.3%)	19359 (33.6%)	12719 (22.1%)
2006	Survivor, with morbidity	18839	909 (1186)	11959 (63.5%)	6672 (35.4%)	6527 (34.6%)	5640 (29.9%)
2006	Deceased	6023	2190 (2833)	3068 (50.9%)	1426 (23.7%)	1922 (31.9%)	2675 (44.4%)
2011	All	66175	751 (1453)	43094 (65.1%)	29785 (45%)	22413 (33.9%)	13977 (21.1%)
2011	Survivor, no morbidity	42440	386 (674)	27733 (65.3%)	21391 (50.4%)	14089 (33.2%)	6960 (16.4%)
2011	Survivor, with morbidity	16261	991 (1079)	11051 (68%)	6511 (40%)	5738 (35.3%)	4012 (24.7%)
2011	Deceased	7474	2296 (3210)	4310 (57.7%)	1883 (25.2%)	2586 (34.6%)	3005 (40.2%)

### Results from regression models

Time trends were further explored within birth cohorts and age classes by use of two-part regression. The full model output is displayed in Table 2. The model results meet common, literature based expectations. In particular, HCE increased with increasing baseline age and calendar year. Moreover, proximity-to-death was also associated with higher costs (for models 2 and 3), as were the morbidity indicators for prior hospitalization, nursing home stays, or high costs (model 3). Moreover, the interaction terms improved the AIC model fit (data not shown). Table 3 offers a different view on the regression results by showing regression model derived HCE predictions. The basic model column illustrates prediction averages for the three birth-cohort groups and four calendar years. Prediction averages increased over calendar years (e.g. from CHF 306 per month in 1997 to CHF 584 in 2011 for the 1931-1935 birth cohort), as well as across the three birth cohorts (e.g. from CHF 306 in 1997 to CHF 502 for the 1921-1925 birth cohort). The remaining columns of Table 3 illustrate how age-and calendar year stratified HCE change after controlling for mortality and morbidity status. For example, monthly HCE in 1997 for the 1931-1935 birth cohort decreased from CHF 306 to CHF 213 after morbidity and mortality adjustments. Of note, these adjustment-driven decreases were even more pronounced for older birth cohorts (e.g. from CHF 944 to CHF 451 for the 1921-1925 birth cohort in 2011).

**Table 2:** Regression results from two-part models

	(1) Basic Model AIC: 18332417				(2) Mortality-adjusted Model AIC: 18033849				(3) Morbidity- and Mortality-adjusted Model AIC: 17781023			
	logit estimate	t-value	glm estimate	t-value	logit estimate	t-value	glm estimate	t-value	logit estimate	t-value	glm estimate	t-value
Female sex	0.293^***^	-25.04	-0.202^***^	-29.08	0.308^***^	-11	-0.108^***^	-9.17	0.341^***^	-12.25	-0.0887^***^	-10.36
Age at baseline												
Age at baseline per year increase	0.0551^***^	-14.55	0.0346^***^	-15.01	0.0550^***^	-7.42	0.0284^***^	-9.12	0.0533^***^	-7.21	0.0293^***^	-11.61
Age at baseline ^2^	0.000355	-1.58	-0.0000115	-0.1	0.0000633	-0.12	-0.00022	-1.2	-0.000202	-0.38	-0.000371^**^	-2.75
Int. age at baseline # female sex	-0.00657^***^	-3.75	0.00926^***^	-10.03	-0.00467	-1.15	0.0127^***^	-8.79	-0.00734	-1.81	0.00825^***^	-7.75
Calendar year												
1997	Ref.				Ref.				Ref.			
1998	0.110^***^	-4.68	0.0412^*^	-2.29	0.107^***^	-6.46	0.0300^*^	-2.13	0.104^***^	-6.02	0.0235	1.47
1999	0.246^***^	-10.07	0.0694^***^	-3.84	0.246^***^	-13.54	0.0638^***^	-4.21	0.238^***^	-12.78	0.0479^**^	-3.05
2000	0.410^***^	-16.12	0.0443^*^	-2.46	0.409^***^	-20.69	0.0480^**^	-3.14	0.392^***^	-19.42	0.0225	-1.44
2001	0.417^***^	-16.23	0.0663^***^	-3.68	0.414^***^	-20.39	0.0592^***^	-3.84	0.386^***^	-18.6	0.0289	-1.82
2002	0.291^***^	-11.64	-0.122^***^	-6.73	0.290^***^	-14.29	-0.145^***^	-8.93	0.257^***^	-12.4	-0.189^***^	-11.45
2003	0.671^***^	-24.2	0.151^***^	-8.34	0.669^***^	-28.72	0.157^***^	-9.65	0.677^***^	-28.66	0.178^***^	-10.86
2004	0.801^***^	-27.59	0.208^***^	-11.43	0.799^***^	-32.01	0.199^***^	-12.34	0.751^***^	-29.58	0.142^***^	-8.82
2005	0.925^***^	-30.53	0.233^***^	-12.79	0.924^***^	-35.03	0.213^***^	-12.86	0.871^***^	-32.61	0.146^***^	-8.74
2006	1.004^***^	-31.9	0.298^***^	-16.21	1.000^***^	-35.91	0.276^***^	-16.53	0.952^***^	-33.72	0.215^***^	-12.92
2007	1.129^***^	-34.14	0.366^***^	-19.87	1.125^***^	-38.03	0.335^***^	-20.33	1.081^***^	-36.07	0.287^***^	-17.45
2008	1.300^***^	-36.68	0.387^***^	-20.91	1.297^***^	-40.08	0.361^***^	-21.66	1.241^***^	-37.88	0.301^***^	-17.78
2009	1.382^***^	-37.33	0.455^***^	-24.32	1.383^***^	-40.79	0.392^***^	-23.83	1.325^***^	-38.57	0.324^***^	-19.82
2010	1.485^***^	-37.98	0.452^***^	-24.02	1.481^***^	-41.04	0.410^***^	-24.75	1.420^***^	-38.9	0.341^***^	-20.55
2011	1.593^***^	-38.66	0.525^***^	-27.69	1.585^***^	-41.09	0.457^***^	-27.54	1.527^***^	-39.07	0.403^***^	-24.27
Int. age at baseline & calendar year												
1998 # Age at baseline	0.000516	-0.16	-0.00227	-1	0.000259	-0.11	-0.00153	-0.91	-0.000105	-0.04	-0.00178	-0.93
1999 # Age at baseline	0.00759^*^	-2.19	-0.000617	-0.27	0.00739^**^	-2.86	-0.000701	-0.39	0.00646^*^	-2.45	-0.00162	-0.84
2000 # Age at baseline	0.00806^*^	-2.22	0.00232	-1.02	0.00721^*^	-2.54	0.00104	-0.56	0.00555	-1.91	-0.000218	-0.11
2001 # Age at baseline	0.0181^***^	-4.83	-0.000646	-0.28	0.0171^***^	-5.68	-0.000981	-0.53	0.0148^***^	-4.83	-0.00354	-1.84
2002 # Age at baseline	0.0135^***^	-3.72	-0.000105	-0.05	0.0121^***^	-4.08	-0.00117	-0.6	0.00922^**^	-3.04	-0.00398^*^	-1.97
2003 # Age at baseline	0.0156^***^	-3.82	-0.000623	-0.27	0.0135^***^	-3.88	-0.00201	-1.03	0.0121^***^	-3.43	-0.00363	-1.83
2004 # Age at baseline	0.0178^***^	-4.11	-0.00119	-0.51	0.0157^***^	-4.1	-0.00127	-0.65	0.0123^**^	-3.18	-0.00459^*^	-2.29
2005 # Age at baseline	0.0146^**^	-3.22	-0.000473	-0.2	0.0118^**^	-2.9	-0.00159	-0.8	0.00715	-1.74	-0.00606^**^	-2.96
2006 # Age at baseline	0.0193^***^	-4.01	0.00219	-0.92	0.0157^***^	-3.6	0.000028	-0.01	0.0107^*^	-2.4	-0.00460^*^	-2.26
2007 # Age at baseline	0.0175^***^	-3.43	-0.000538	-0.22	0.0138^**^	-2.96	-0.00224	-1.12	0.00842	-1.78	-0.00760^***^	-3.77
2008 # Age at baseline	0.0157^**^	-2.83	0.00281	-1.16	0.0107^*^	-2.07	-0.000687	-0.34	0.0045	-0.86	-0.00518^*^	-2.49
2009 # Age at baseline	0.0170^**^	-2.89	0.00357	-1.44	0.0128^*^	-2.33	-0.00128	-0.63	0.00498	-0.89	-0.00694^***^	-3.37
2010 # Age at baseline	0.0198^**^	-3.12	0.00625^*^	-2.48	0.0145^*^	-2.41	-0.00153	-0.75	0.00622	-1.02	-0.00765^***^	-3.68
2011 # Age at baseline	0.0161^*^	-2.37	0.00374	-1.46	0.00892	-1.35	-0.00219	-1.07	-0.000527	-0.08	-0.00915^***^	-4.36
Survival status												
Death in same calendar year					1.490^***^	-10.27	2.562^***^	-89.94	1.154^***^	-7.62	2.304^***^	-71.74
Death in next calendar year					0.830^***^	-7.52	1.704^***^	-55.97	0.477^***^	-4.26	1.516^***^	-46.82
Survivor					Ref.				Ref.			
Int. survival status # age at baseline												
Death in same calendar year # Age at baseline					-0.00624	-0.66	-0.0560^***^	-37.21	-0.00807	-0.84	-0.0452^***^	-27
Death in next calendar year # Age at baseline					0.0108	-1.49	-0.0463^***^	-34.1	0.0101	-1.37	-0.0400^***^	-25.95
Int. calendar year # survival status												
1998 # Death in same calendar year					0.24	-1.25	-0.0431	-1.21	0.225	-1.16	-0.035	-0.87
1998 # Death in next calendar year					0.0686	-0.48	-0.0212	-0.51	0.0634	-0.44	-0.0215	-0.5
1999 # Death in same calendar year					0.0198	-0.1	-0.0682	-1.91	-0.0202	-0.11	-0.0662	-1.74
1999 # Death in next calendar year					-0.264	-1.93	-0.0658	-1.66	-0.291^*^	-2.09	-0.0393	-0.94
2000 # Death in same calendar year					0.0206	-0.1	-0.140^***^	-4.02	-0.0131	-0.07	-0.122^**^	-3.27
2000 # Death in next calendar year					0.029	-0.19	-0.0541	-1.44	-0.0419	-0.28	-0.106^**^	-2.69
2001 # Death in same calendar year					-0.0107	-0.05	-0.158^***^	-4.61	-0.119	-0.6	-0.192^***^	-5.1
2001 # Death in next calendar year					0.0993	-0.64	-0.137^***^	-3.61	0.0289	-0.18	-0.193^***^	-4.95
2002 # Death in same calendar year					0.214	-1.07	-0.0730^*^	-2.12	0.0921	-0.46	-0.0944^*^	-2.44
2002 # Death in next calendar year					-0.19	-1.4	-0.242^***^	-6.23	-0.242	-1.74	-0.289^***^	-6.94
2003 # Death in same calendar year					0.0375	-0.18	-0.332^***^	-9.23	0.0281	-0.13	-0.296^***^	-7.81
2003 # Death in next calendar year					0.0587	-0.37	-0.224^***^	-6.09	0.0712	-0.44	-0.244^***^	-6.31
2004 # Death in same calendar year					0.143	-0.64	-0.341^***^	-10.55	-0.0105	-0.05	-0.348^***^	-9.35
2004 # Death in next calendar year					-0.158	-1.02	-0.270^***^	-7.62	-0.217	-1.38	-0.312^***^	-8.22
2005 # Death in same calendar year					-0.169	-0.82	-0.331^***^	-9.83	-0.323	-1.56	-0.324^***^	-8.47
2005 # Death in next calendar year					-0.00632	-0.04	-0.274^***^	-7.59	-0.0598	-0.36	-0.307^***^	-7.87
2006 # Death in same calendar year					0.18	-0.77	-0.361^***^	-10.67	0.0315	-0.13	-0.350^***^	-9.09
2006 # Death in next calendar year					-0.0632	-0.38	-0.311^***^	-8.77	-0.157	-0.94	-0.339^***^	-8.94
2007 # Death in same calendar year					0.00000638	0	-0.418^***^	-12.94	-0.151	-0.66	-0.382^***^	-10.43
2007 # Death in next calendar year					-0.208	-1.3	-0.321^***^	-9.24	-0.283	-1.74	-0.347^***^	-9.22
2008 # Death in same calendar year					-0.164	-0.74	-0.484^***^	-14.83	-0.337	-1.51	-0.477^***^	-12.93
2008 # Death in next calendar year					-0.0271	-0.15	-0.339^***^	-9.86	-0.133	-0.75	-0.372^***^	-10.02
2009 # Death in same calendar year					-0.879^***^	-4.77	-0.335^***^	-7.86	-1.061^***^	-5.68	-0.329^***^	-6.87
2009 # Death in next calendar year					-0.0265	-0.15	-0.407^***^	-12.04	-0.116	-0.64	-0.413^***^	-11.41
2010 # Death in same calendar year					-0.745^***^	-3.83	-0.427^***^	-12.98	-0.919^***^	-4.66	-0.386^***^	-10.32
2010 # Death in next calendar year					0.0311	-0.17	-0.454^***^	-13.67	-0.0672	-0.36	-0.466^***^	-13.14
2011 # Death in same calendar year					-0.610^**^	-3.01	-0.469^***^	-13.9	-0.763^***^	-3.74	-0.422^***^	-10.82
2011 # Death in next calendar year					0.167	-0.84	-0.488^***^	-14.76	0.0776	-0.39	-0.486^***^	-13.64
Morbidity indicators yes/no												
Hospitalization in prior year									1.491^***^	-47.65	0.651^***^	-74.7
Hospitalization in prior year # Age at Baseline									0.0110^*^	-2.48	-0.00777^***^	-7.65
High outpatient drug expenditures in prior year									4.086^***^	-33.7	0.893^***^	-131.66
Nursing home stay in prior year									3.090^***^	-26.93	0.841^***^	-125.92
Int. hospitalization # survival status												
Hospitalization in prior year # Death in same calendar year									-0.870^***^	-9.12	-0.250^***^	-16.96
Hospitalization in prior year # Death in next calendar year									0.653^***^	-4.36	-0.214^***^	-16.59
Constant	1.460^***^	-80.67	5.983^***^	-435.25	1.433^***^	-57.82	5.772^***^	-367.05	1.272^***^	-51.26	5.543^***^	-383.59

^*^p < 0.05, ^**^p < 0.01, ^***^p < 0.001

**Table 3:** Regression model-derived HCE estimations (based on the regression models shown in Table 2) per birth cohort and year. Estimates represent HCE per month in Swiss Francs, as well as bootstrapped 95% Confidence Intervals in square brackets

Birth Cohort	Year	Age classes	(1) Basic Model	(2) Mortality adjusted	(3) Mortality & morbidity-adjusted	(4) Mortality & PCG-adjusted (2006 and later)
1931-1935	1997	61-65	306 [298;314]	270 [264;276]	213 [208;217]	-
1931-1935	2001	66-70	343 [335;352]	300 [294;306]	229 [225;233]	-
1931-1935	2006	71-75	453 [443;464]	389 [382;397]	288 [283;294]	163
1931-1935	2011	76-80	584 [570;597]	475 [466;484]	354 [348;361]	213
1926-1930	1997	66-70	393 [386;400]	338 [333;344]	260 [256;264]	-
1926-1930	2001	71-75	437 [430;445]	373 [367;379]	274 [271;278]	-
1926-1930	2006	76-80	579 [569;588]	480 [473;487]	339 [335;344]	197
1926-1930	2011	81-85	744 [732;757]	574 [566;583]	404 [398;410]	245
1921-1925	1997	71-75	502 [489;514]	417 [408;426]	310 [304;317]	-
1921-1925	2001	76-80	553 [541;564]	455 [445;464]	320 [314;326]	-
1921-1925	2006	81-85	734 [720;749]	582 [570;594]	390 [382;398]	233
1921-1925	2011	86-90	944 [922;966]	684 [670;698]	451 [441;461]	286

**Fig. 5:**
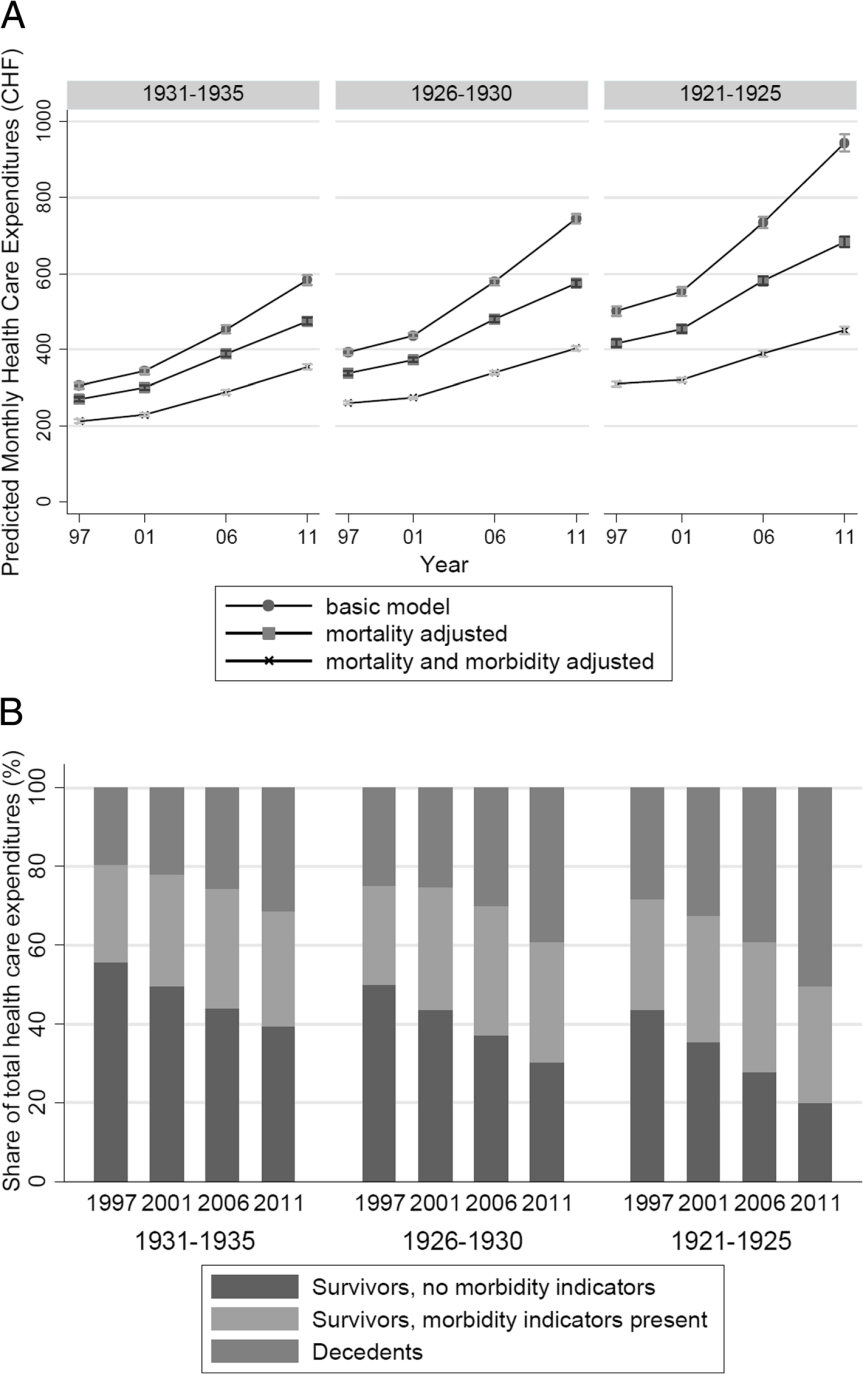
Panel A: Birth cohort analysis, by baseline age classes and calendar year. HCE are model-based, age-associated predictions from different two-part models adjusted for age and sex (basic model), survivor status (mortality adjusted), as well as survivor status and morbidity indicators. Panel B: Share of different groups (by survivor and morbidity status) on overall HCE volume, by birth cohort (outer x-axis) and calendar year (inner x-axis). HCE estimates are derived from a two-part regression model adjusted for sex, survivor-status, and morbidity status.

Figures 5a (birth cohort) and 6a (age class) plot HCE estimates over calendar time per birth cohort or age class. In the birth cohort analysis, there was a widening gap between basic and survival-adjusted estimates because basic estimates grew at a higher rate (which implies a rising importance of proximity-to-death on age-specific HCE averages). Moreover, by applying results from the morbidity and mortality-adjusted model, Figure 5b illustrates the share of three groups of insured (deceased, survivors with morbidity indicators, and survivors without morbidity indicators) of overall HCE volume in a given birth cohort and year. The bars show an increasing importance of decedents for overall HCE over time, as well as with increasing age. In particular, the percentage of proximity-to-death related HCE increased from 19% to 31% for the 1931-1935 birth cohort, from 25% to 39% for the 1926-1930 birth cohort, and from 28% to 51% for the 1921-1925 birth cohort, respectively. By contrast, the percentage of HCE stemming from persons with morbidity indicators remained more constant across birth cohort and time, ranging from 25% to 33%.

**Fig. 6:**
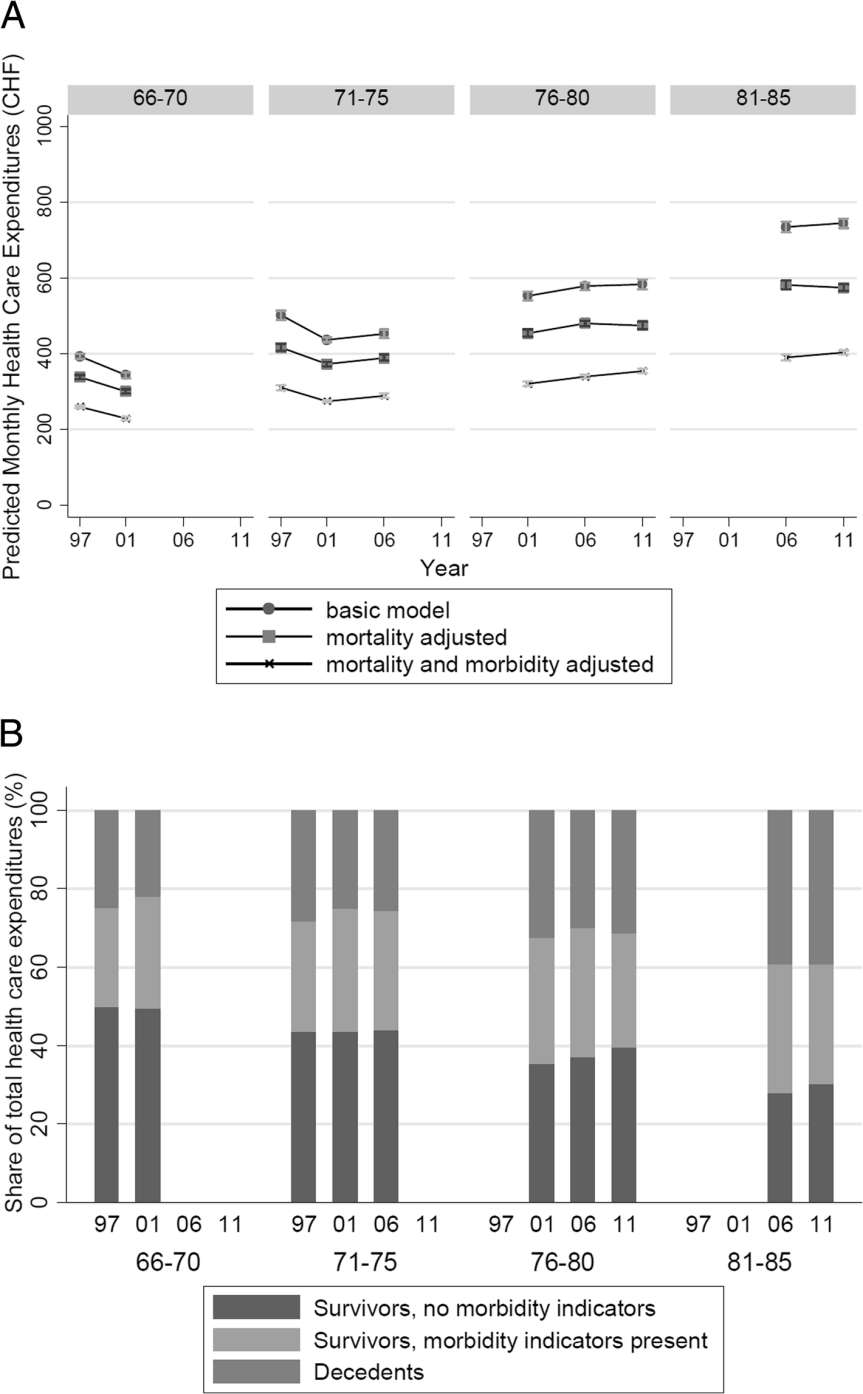
Panel A: Age class analysis, by calendar year. HCE model-based, age class-associated predictions from different two-part models adjusted for age and sex (basic model), survivor status (mortality adjusted), as well as survivor status and morbidity indicators. Panel B: Share of different groups (by survivor and morbidity status) on overall HCE volume by age class (outer x-axis) and calendar year (inner x-axis). HCE estimates are derived from a two-part regression model adjusted for sex, survivor-status, and morbidity status.

The age class analyses displayed in Figure 6a showed less HCE variability across calendar time. The younger age classes exhibited both decreases and increases (e.g. from CHF 502 per month to CHF 453 for the age class 71-75 between 1997 and 2006 in the basic model), whereas average HCE in the classes aged 76-80 and above were steadily increasing over time (e.g. from CHF 553 to CHF 584 for the age class 76-80 between 2001 and 2011 in the basic model). Along the same lines, shifts in cost composition owing to mortality and morbidity were not in one single direction and relatively small when compared to results from the birth cohort analyses (Figure 6b). Nevertheless, the age class analysis also provided some indication that mortality has declined over time. As illustrated by Table 4, the population share of deceased persons declined slightly over calendar time for all age classes (e.g. by -3.3%-points between 2006 and 2001 for the age class 81-85).

**Table 4:** Regression-derived changes in HCE volume and number of individuals across different age classes over time, stratified by survivor and morbidity status. All estimates were derived from the mortality- and morbidity-adjusted two-part regression model

Age Class	Subgroup	Comparator groups				Population size change			HCE volume change (CHF)			HCE change rates (per year)	
		time point 1	birth cohort	time point 2	birth cohort	time point 1	time point 2	change	HCE volume time point 1	HCE volume time point 2	change	crude	population change corrected ^a^
66-70	Overall	1997	1926-30	2001	1931-35	34758	36554	105.2%	14437510	13464464	93.3%	-1.7%	-3.0%
71-75	Overall	1997	1921-25	2006	1931-35	30935	33582	108.6%	16279928	16682277	102.5%	0.3%	-0.6%
76-80	Overall	2001	1921-25	2011	1931-35	27001	29785	110.3%	16561328	19265326	116.3%	1.5%	0.5%
81-85	Overall	2006	1921-25	2011	1926-30	21034	22413	106.6%	17906461	18901719	105.6%	1.1%	-0.2%
66-70	Survivors, morb.	1997	1926-30	2001	1931-35	5766	6328	109.7%	3675239	3826840	104.1%	1.0%	-1.3%
71-75	Survivors, morb.	1997	1921-25	2006	1931-35	6123	6672	109.0%	4538328	5038134	111.0%	1.2%	0.2%
76-80	Survivors, morb.	2001	1921-25	2011	1931-35	6454	6511	100.9%	5330491	5653214	106.5%	0.6%	0.5%
81-85	Survivors, morb.	2006	1921-25	2011	1926-30	5640	5738	101.7%	5901028	5787366	98.1%	-0.4%	-0.7%
66-70	Decedents	1997	1926-30	2001	1931-35	1375	1144	83.2%	3580834	2975086	83.1%	-4.5%	0.0%
71-75	Decedents	1997	1921-25	2006	1931-35	1897	1426	75.2%	4634687	4293761	92.6%	-0.8%	2.3%
76-80	Decedents	2001	1921-25	2011	1931-35	2222	1883	84.7%	5363521	6030700	112.4%	1.2%	2.9%
81-85	Decedents	2006	1921-25	2011	1926-30	2675	2586	96.7%	7045892	7423799	105.4%	1.1%	1.7%

^a^Population change corrected means that HCE volumes for time point 2 were weighted by the population size change occurring between time points 1 and 2 (in order to emulate equal population sizes).

Because the morbidity indicators employed in this analysis are known to primarily address severe illnesses, the major findings were subjected to a sensitivity analysis including pharmaceutical cost groups as additional morbidity markers (which were only available for the calendar years between 2006 and 2011, however). The re-estimated mortality- and morbidity-adjusted model with additional PCG-adjustments further decreased HCE estimates for “morbidity-free”, surviving individuals. Relative to the morbidity- and mortality-adjusted estimates presented in Table 3, the PCG-adjusted model (last column) led to further decreases, for example from CHF 288 to CHF 163 (43% reduction, 1931-1935 birth cohort in 2006) or from CHF 451 to CHF 286 (37% reduction, 1921-1925 birth cohort in 2011). Moreover, the inclusion of PCG led to many more persons being classified as having a chronic illness (74% individuals in 2006 instead of 27% in the original analysis). This was expected because the morbidity indicators employed in the main analysis were primarily indicative of severe illnesses requiring inpatient stays but tended to miss manageable, but prevalent chronic diseases like diabetes mellitus. This is also illustrated by a comparison of HCE. In 2006, persons with a PCG-indicated morbidity had an average HCE accrual of CHF 422 per month as opposed to CHF 909 in the group with morbidity indicators as defined for the main analysis.

### Health care expenditure growth rates across age classes

A further aim was to compare HCE growth rates over time across different age classes. Table 4 illustrates total HCE volume and population size changes for four age classes between 66 and 85 years of age according to survival and morbidity status. The main information is located in the last two columns of Table 4. Crude yearly HCE change rates were derived from age class specific HCE volume differences between the two time points but did not account for changes in population size. Similar to HCE estimates presented in Figure 6a, overall growth rates were negative (-1.7%) or slightly positive (0.3%) for the two younger age classes and exceeding the value of 1% per year for the two older age classes (1.5% and 1.1%, respectively). Further noteworthy, in decedents, the adjustment of growth rates for population size changes substantially increased growth estimates (e.g. from -0.8% to 2.3% for decedents from the 71-75 age class). This suggests that, keeping group sizes constant, the HCE volume for decedents of the same age class rose with increasing calendar time (with the exception of the 66-70 age class, where population change adjusted growth was 0%). Of note, this increase is on top of the general deflation applied to all HCE. By contrast, overall age class-specific HCE volume increases were small or even negative when considering the full sample (i.e. including decedents and survivors), reinforcing the notion that HCE growth is not homogenous across all age classes of the elderly.

## Discussion

Using a panel dataset of 104,000 Swiss elderly insured, this study analyzed HCE evolution along two different age dimensions (birth cohorts and age classes) to explore the impact of proximity to death on HCE growth over time. The first analysis dimension focused on longitudinal cost growth within three birth cohorts, thereby exploring the effect of “aging” viz. proximity-to-death and morbidity. As expected HCE increased non-linearly with rising age, even when adjusting for imminent death (that is, a remaining lifetime of <2 years from a given calendar year). Moreover, the HCE volume share of persons close to death increased markedly as age and calendar time progressed, ranging from 19% to 51%. This finding implies that proximity-to-death is a major, but not the only driver of HCE growth with rising age. Indeed, results from morbidity-adjusted analyses also pointed to the considerable role of chronic and hospital-treated illnesses as important factors. An exact quantification of the impact of morbidities remains difficult however, as this depends on availability of diagnostic information and morbidity definitions. For example, when compared with relatively crude, but time-consistent morbidity indicators based on hospitalizations and high drug expenditures, a PCG-augmented sensitivity analysis almost tripled the share of persons with morbidities from one in four to nearly three in four. What is more, the effect of morbidity was intertwined with proximity to death, which may have led to a general over-interpretation of the role of mortality on HCE growth. [17] Indeed, the PCG-based sensitivity analysis (including persons aged 71 and older in 2006) revealed that 90% of decedents and 73% of survivors had PCG-based morbidity indicators (compared with 59% and 25%, respectively, when applying hospitalization and drug cost-based indices).

A second analysis looked into the evolution of HCE for select age classes over time (e.g. all individuals aged 71-75 years in 1997, 2001, and 2006). Interestingly, cost growth patterns were not unidirectional, and analyses for younger age classes even suggested decreases over time. However, as all analyses were performed on deflated HCE (using nationwide HCE growth among 19-60 year old persons as a deflator), these results merely suggested that there was no consistent surplus HCE growth among elderly within the same age class over time. Analyses by other groups have found considerable cost profile steepening by age, that is, higher HCE increases over time for older age classes. [26] The findings from the age class analysis supported this notion of steepening for the two oldest age classes, but not to the extent reported by others. However, it is possible that actual steepening was underestimated by this study because mandatory health insurance only accounts for 35% of all HCE in Switzerland. For example, the share of nursing home costs covered by social health insurance is only around 18% and limited to medical care, not including hospitality and other services.

Overall, the findings from this study fell in line with the majority of other analyses reporting a marked impact of proximity-to-death on HCE growth, particularly those that have also explored the role of morbidity by use of various indicators. [4, 10, 17, 27] One of the most detailed analyses was performed by Wong and colleagues, who found that proximity-to-death even holds within subgroups of persons affected by 93 lethal and non-lethal diseases, but with non-negligible age effects [27, 28] The wide inpatient HCE variability between diseases suggested that HCE projections should also take the changing prevalence of specific diseases into account. [17, 29] Moreover, with its population focus, the present work resembles the analyses by Seshamani & Gray [11] who (and others [13]) also did not observe continuous HCE growth among decedents. Their estimated shares of decedent costs ranged from 19% to 31% and were therefore similar to the ones observed in this analysis (which, however, covered a longer and more recent time period).

This work contributes to the ongoing debate on age-related HCE drivers by analyzing data over a longer time period than most other studies and by introducing novel study design aspects, namely the simultaneous investigation of longitudinal HCE growth within contemporary birth cohorts and across age classes over different years. The availability of a large panel data set with complete mortality information, combined with the chosen analytic strategy allowed consistent control over a number of possible confounders (migration, sex or health status), which may have affected other cross sectional based studies. Novel findings of this study concerned the dynamics of HCE volumes attributable to persons with imminent death, which indeed tended to rise with increasing age. The present analysis also yielded indications that, within the same age class, the share of persons with imminent death decreased over time. Of note, this finding was unlikely to be caused by survivor bias because of the panel design of this analysis and complete data on survival status. By contrast, the lack of morbidity indicators hampered insights into further drivers for cost growth that were still subsumed under age-associated effects in this analysis. Moreover, this study did not specifically address the prevalent endogeneity problem between health care expenditures and remaining lifetime, and observed associations likely do not have a causal interpretation. [7]

## Conclusion

To conclude, this analysis pointed to a major, but not exclusive role of proximity-to-death on HCE growth among elderly. Falling in line with recent research, this study confirmed that morbidity was a key factor pushing HCE growth with rising age and that enhanced health status indicators will be key to a better understanding of age and “healthy aging” on overall HCE growth. These results suggest that, given the residual age-associated HCE growth observed in this study and the expected demographic shift towards more elderly, further, demography-related HCE growth is likely. Because of the relevance of chronic morbidities in the observed sample and the elderly population at large, potential remedies to dampen the expected HCE increases may include more efficient management of chronic illnesses and potentially earlier and wider application of palliative care. However, such measures should not solely focus on elderly persons but include younger age classes, which are also substantial contributors to overall HCE growth and health care utilization expansion.

## Additional file

Additional file 1: **Figure S1.** Kaplan-Meier survival curve by birth cohort. The year 0 corresponds to 1996. (DOCX 731 kb)

## Abbreviations

AIC: Aikaike’s Information Criterion
CHF: Swiss Francs
GLM: Generalized Linear Model
HCE: Health Care Expenditures
PCG: Pharmaceutical Cost Groups
