# Supplementary material for: Proximity to death and health care expenditure increase revisited: A 15-year panel analysis of elderly persons
Source: Health Econ Rev. 2019 Mar 11;9:9. doi: 10.1186/s13561-019-0224-z (PMC6734245; doi:10.1186/s13561-019-0224-z)

Supplementary Materials

Supplementary Figure 1: Kaplan-Meier survival curve by birth cohort. The year 0 corresponds to 1996.


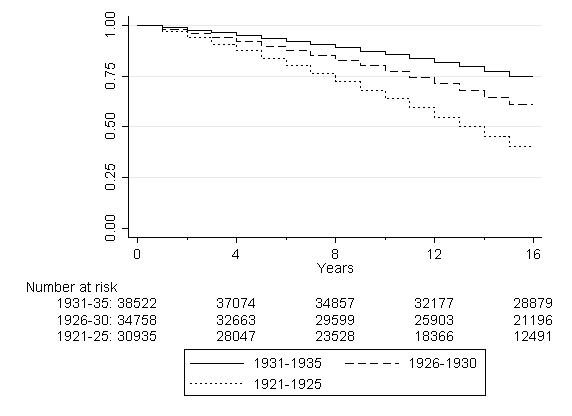

Supplement: Supplementary file 1 — Figure S1. Kaplan-Meier survival curve by birth cohort. The year 0 corresponds to 1996. (DOCX 731 kb) [file 13561_2019_224_MOESM1_ESM.docx]
